# 2316 The extra-territorial translational team: Advances in multi-faceted community engagement

**DOI:** 10.1017/cts.2018.235

**Published:** 2018-11-21

**Authors:** Sharon A. Croisant, Amber L. Anthony, John Prochaska, Chantele Singleton, Joseph A. Kotarba

## Abstract

OBJECTIVES: We developed the concept of the *extra-territorial translational team (ETTT)* in 2014 as a more inclusive revision and extension of the *team science* concept. Translational thinking is largely marked by the perception of the team as a thing-like structure at the center of the scientific activity. Collaboration accordingly involves bringing external others (e.g., scientists, community members, and clinicians) into the team through limited or dependent participation. The ETTT is intended to frame the team as an *idea*: a schema for assembling and managing *relationships* among otherwise disparate individuals with vested interests in the problem at hand. Thus, the ETTT can be seen as a *process* as well as an *object*. Our initial focus was on the very successful *SCI Café* program (where Science and Communities Interact) conducted through the Institute for Translational Sciences and the Center for Translational Sciences Award at UTMB. We found that by looking beyond the taken-for-granted features of translational research teams, we are free to discover new ways of organizing research and community engagement that are innovative yet productive. The major area of growth, however, has been the Research, Education, And Community Health Coalition (REACH). The purpose of the current study is to outline strategies for inventorying and evaluating the emerging programs that are the major components of REACH and the SCI Café and to suggest implications for the extra-territorial translational team concept. METHODS/STUDY POPULATION: The assessment of the extraterritorial team concept in REACH and SCI Café is primary a process of qualitative content analysis. We use semi-structured interviews with project leadership, observations of the actual performance of the REACH teams, and the review of REACH and SCI Café documents, for example, Quantitatively, we have conducted a Community Health Needs Assessment (CHNA) to better understand community health and resource needs. RESULTS: Both the SCI Café program and the REACH initiative follow the principles of the ETTT concept for assembling and managing research and community outreach. The following are several key principles shared by both programs: (1) The importance of creative, applicable, and inclusive mission statements: (a) REACH seeks to facilitate communication, collaborative research, and service efforts between UTMB and Institute for Translational Sciences investigators and Galveston County community leaders; (b) The SCI Café hosts interactive dialogs that serve as a medium for priming, organizing, communicating and strategizing among the individuals involved in team science via community-based research projects. (2) Increasing scientific and health literacy: (a) REACH seeks to increase literacy through both short-term and long-term interactions; (b) The SCI Café focuses on short-term yet intensive interaction through conversations among researchers, clinicians, and the public. (3) Sharing timely scientific public health information with the community: (a) REACH seeks information from community leaders on relevant topics; (b) The SCI Café can mobilize quickly to respond to timely topics by direct communication with a wide range of stakeholders, academic as well as community based. (4) Sharing leadership with the community: (a) REACH establishes formal relationships with 23 UTMB units and 39 broad-based, high impact Galveston County organizations. (b) The SCI Café works primarily with “grass roots” community-level groups and organizations. (5) Creating resources and strategies for expansion: (a) REACH is working to expand its activities to other counties in the Gulf Coast area of Texas (e.g., Brazoria and Matagorda Counties); (b) The SCI Café is expanding its program to comfortable locations accessible to local residents (e.g., schools and libraries). (6) The value of regular and systematic scientific and evaluation: (a) REACH is conducting a Community Health Needs Assessment (CHNA) that has already discovered major issues of relevance to community leaders including mental health, vaccination rates, food security, disaster preparedness, and caregiving. (b) The SCI Café conducts an evaluation survey at the conclusion of every event to stay current with participants interests and needs. DISCUSSION/SIGNIFICANCE OF IMPACT: (1) In order to maintain the ability to operate extra-territorially (i.e., beyond the safe organizational confines of the University), the 2 programs discussed here must maintain a fluid team structure. Different projects require different types of leadership, grass roots participation, university resources, communications/public relations, etc. (2) The strategy of accumulating and disseminating best practices appears to be one of the most valuable products of the extra-territorial team. (a) REACH’s “Offer and Ask” practice by which information of university and community resources (skills and expertise) are shared makes cooperation and shared leadership explicit. (b) The SCI Café’s interactional strategies for encouraging and enabling café participants to join the discussion/conversation are wonderful ways to convert an otherwise unidirectional lecture into a vibrant conversation. (3) Although the scope of these 2 programs is quite different, the message from both is that the principles of extra-territorial translational teams are application to all such endeavors to improve scientific and health literacy.

